# *IFITM3* Polymorphism rs12252-C Restricts Influenza A Viruses

**DOI:** 10.1371/journal.pone.0110096

**Published:** 2014-10-14

**Authors:** David Evan Joseph Williams, Wan-Lin Wu, Christopher Robert Grotefend, Vladimir Radic, Changik Chung, Young-Hwa Chung, Michael Farzan, I-Chueh Huang

**Affiliations:** 1 Department of Cell Biology and Neuroscience, College of Natural and Agricultural Sciences, University of California Riverside, Riverside, California, United States of America; 2 Department of Infectious Diseases, The Scripps Research Institute, Jupiter, Florida, United States of America; 3 BK21+, Department of Cogno-Mechatronics Engineering, Pusan National University, Busan, Republic of Korea; University of Pittsburgh, United States of America

## Abstract

The *IFITM3* polymorphism rs12252-C, which encodes an IFITM3 isoform (Δ21 IFITM3) lacking 21 amino acids at the amino terminus, has been controversially associated with poor clinical outcomes in patients with H1N1 influenza A virus (IAV) infections. *In vitro* studies have shown that Δ21 IFITM3 loses its ability to restrict H1N1 IAV. Subsequent research has also revealed that tyrosine 20 is the key determinant for IFITM3 endocytic trafficking, which is essential for the efficient anti-viral activity of IFITM3. In contrast to previous studies, we demonstrated that both Δ21 IFITM3 and an IFITM3 variant (Y20A IFITM3), in which tyrosine 20 is substituted with alanine, strongly restricted entry mediated by IAV H1, H3, H5, and H7 proteins. Δ21 IFITM3 also efficiently suppressed replication of H1N1 and, to a lesser extent, H3N2 IAV. Δ21 IFITM3 and Y20A IFITM3 had broader subcellular distributions than full-length IFITM3 but an abundant amount of both IFITM3 variants still localized to late endosomes and lysosomes. Our data indicate that tyrosine 20 partially regulates the subcellular localization of IFITM3 but is not functionally essential for IFITM3-mediated IAV restriction. They also suggested that mechanisms, other than viral entry restriction, might contribute to variations in clinical outcomes of H1N1 influenza associated with rs12252-C.

## Introduction

Interferon-induced transmembrane (IFITM) proteins, including IFITM1, 2, and 3, restrict a broad range of highly pathogenic human viruses [1]–[5]. Among them, IFITM3 plays a central role in limiting influenza A virus (IAV) entry and replication *in vitro* and *in vivo* [6], [7]. Recent studies have shown that *IFITM3* polymorphism rs12252-C, which encodes an aberrant form of IFITM3 lacking the amino-terminal (N-terminal) 21 amino acids (Δ21 IFITM3), contributes to poor clinical outcomes in patients with H1N1 IAV infections [7], [8]. However, the association between rs12252-C polymorphism and the severity of H1N1 influenza could not be established in research analyzing more than 5000 subjects in two separate cohorts [9]. The rs12252-C polymorphism has also been reported to be a prognostic factor for H7N9 avian influenza. rs12252-C carriers developed more severe symptoms and signs, had higher viral and cytokine titers, and experienced higher mortality [10]. Although the prevalence of *IFITM3* polymorphism rs12252-C is extremely low among Caucasians, the 1000 Genomes Project has revealed a high prevalence of rs12252-C in East Asian populations—most substantially in the Japanese population, with an allele frequency of 43.82% being homozygous carriers, and, to a lesser extent, in the Han Chinese population, with a homozygous allele frequency of 25.38% [7], [8].

The underlying mechanism of the poor prognosis in rs12252-C carriers after IAV infection is still not clear. Everitt et al. observed that Δ21 IFITM3 lost its ability to restrict H1N1 IAV replication *in vitro*. However, they could not detect expression of Δ21 IFITM3 in lymphoblastoid cell lines (LCL) homozygous for rs12252-C at the protein level [7]. In addition to IAV, subsequent studies showed that Δ21 IFITM3 was incapable of inhibiting vesicular stomatitis virus (VSV) and that tyrosine 20 of IFITM3 was critical for IFITM3 subcellular localization and its anti-viral function [11]–[14]. Replacing tyrosine with alanine (Y20A IFITM3) shifted late endosome/lysosome-associated IFITM3 to the plasma membrane and severely diminished its effect on viral restriction. Tyrosine 20 was also reported to be a major determinant for regulating IFITM3 ubiqitination and endocytic trafficking [13], [14]. Tyrosine 20-mediated interaction with adaptor protein 2 (AP2) contributed to IFITM3 endocytosis, which was thought to be essential for IFITM3-mediated restriction [14]. Although tyrosine 20 is a substrate of tyrosine-protein kinase FYN, depleting the expression of FYN did not impair the function of IFITM3 [13].

Contrary to previous observations, our studies demonstrated that both Δ21 IFITM3 and Y20A IFITM3 restricted IAV entry and replication. Δ21 IFITM3 and Y20A IFITM3 strongly inhibited entry mediated by IAV H1, H5, and H7 (Shan; from A/Shanghai/02/2013 [H7N9]), and, to a lesser extent, by H3 and H7 (FPV; from A/FPV/Rostock/34 [H7N1]) proteins. In parallel, H1N1 IAV replication was suppressed as efficiently by Δ21 IFITM3 as full-length (FL) IFITM3, whereas an attenuated effect of Δ21 IFITM3 on H3N2 IAV replication could be detected. Different from FL IFITM3, which concentrated in the late endosomal/lysosomal compartments, both Δ21 IFITM3 and Y20A IFITM3 distributed at the plasma and late endosomal/lysosomal membranes. Epitope-tagged IFITM3 isoforms, which have been commonly used in IFITM-related research, had inconsistent inhibitory effects on IAV entry and replication. They also had different subcellular distributions from those of native IFITM3 isoforms. Our data indicate that 21 amino acids at the N-terminus of IFITM3 are not critical and that tyrosine 20 of IFITM3 is not a major determinant of IFITM3-mediated IAV restriction. They also suggest that poor clinical prognosis in rs12252-C carriers may not be caused by defective anti-viral activity of Δ21 IFITM3. Finally, because epitope tags alter properties of IFITM3, native IFITM3 isoforms would be better models for IFITM studies.

## Experimental Procedures

### Cells

Human embryonic kidney 293T and African green monkey epithelial Vero E6 cells (American Type Culture Collection) were maintained in Dulbecco's modified eagle medium (DMEM; Invitrogen). Human lung epithelial A549 cells were grown in Roswell Park Memorial Institute (RPMI) 1640 medium (Invitrogen). All media were supplemented with 10% fetal bovine serum (FBS; Invitrogen), 100 U/ml penicillin, and 100 µg/ml streptomycin (Invitrogen). A549 cells transduced with a vector or to express tetracycline (tet)-inducible IFITM3 isoforms were selected with 3 µg/ml puromycin (Invitrogen) and 400 µg/ml gentamicin (Invitrogen).

### Plasmids and constructs

DNA segments encoding native, N-terminal c-myc- or flag-FL IFITM3 and Δ21 IFITM3 sequences were introduced into the pRetroX-Tight-Pur vector (Clontech) using restriction enzymes *Bam*HI and *Eco*RI. DNA fragments encoding native IFITM3 and Δ21 IFITM3 were also cloned into the pQCXIP vector (Clontech) via restriction enzymes *Not*I and *Bam*HI. Y20A IFITM3 was generated using the QuikChange method (Agilent Technologies). Influenza A/Shanghai/02/2013 (H7N9) virus HA gene sequence was synthesized (Invitrogen) and introduced into the pCAGGS vector with restriction enzymes *Not*I and *Xho*I.

### Pseudotyped murine leukemia viruses (MLVs) for transduction and infection assays

Plasmids and procedures used to generate pseudotyped MLV-green fluorescent protein (GFP) and transducing viruses have been previously described [2], [15], [16]. Viral entry glycoproteins used to generate MLV-GFP included influenza A virus hemagglutinin (HA) proteins from A/PR/8/34 (H1N1) (H1[PR]), A/Udorn/72 (H3N2)(H3[Ud]), A/Thailand/2(SP-33)/2004(H5N1)(H5[Thai]), A/FPV/Rostock/34 (H7N1)(H7[FPV]), and A/Shanghai/02/2013 (H7N9) (H7[Shan]), the G protein from vesicular stomatitis virus (VSV, Indiana strain), and the env protein from amphotrophic MLV [15]–[18]. Viral entry glycoproteins used to generate transducing viruses included Machupo virus and lymphocytic choriomeningitis virus glycoproteins (GPCs) [18]. For transduction, cells were incubated with transducing viruses mixed with 10 µg/ml of polybrene (Santa Cruz Biotechnology) and centrifuged at 4°C for 30 minutes at 4,000×g. Transduced cells were maintained in growth medium and employed for antibiotic selection or infection 48 hours later. The procedure for MLV-GFP pseudovirus infection were similar to that for transduction. Spin inoculation was used but polybrene was not included. 48 hours after infection, infected cells were harvested, fixed with 1% formaldehyde (Polysciences), and analyzed by flow cytometry. Student's *t* test was used for statistic analysis. To test the effect of type I interferon (IFN) on viral entry and replication, cells were treated with 1000 U/ml human IFN-β 1A (R&D Systems) 48 hours before infection.

### IAV infection assays

Influenza A/PR/8/34 (H1N1), A/Virginia/ATCC3/2009 (H1N1), and A/England/42/72 (H3N2) viruses purchased from American type culture collection (ATCC) were propagated and titered as described [16]. A549 cells treated with various conditions were incubated with H1N1 viruses at the multiplicity of infection (M.O.I.) of 1 or with influenza A/England/42/72 (H3N2) virus at an M.O.I of 0.5 for 3 hours. After infection, cells were maintained in growth medium and harvested 16 hours after inoculation. Infected cells were labeled with 0.5 µg/ml murine anti-influenza H1 IgG2a (C179; Clontech) or with 1 µg/ml anti-influenza viral H3 IgG1 (F49; Clontech), followed by an R-phycoerythin (PE)-conjugated secondary antibody (Thermo scientific). Fixed cells were analyzed by flow cytometry. Student's *t* test was used for statistic analysis.

### Western blotting

Cells were lysed with 2% lubrol (MP Biomedicals), prepared in reducing buffer, heated at 75°C for 10 minutes, analyzed by sodium dodecyl sulfate polyacrylamide gel electrophoresis (SDS/PAGE), and transferred to a polyvinylidene difluoride membrane (Invitrogen). Expression of c-myc-tagged and flag-tagged IFITM3 isoforms was detected by 0.4 µg/ml murine monoclonal anti-c-myc antibody (9E10; Santa Cruz Biotechnology) and 1 µg/ml murine monoclonal anti-flag antibody (M2; Sigma). A goat anti-IFITM2/3 antibody (1 µg/mL; R&D Systems [Cat. AF4834]) and a rabbit anti-IFITM1/2/3 antibody (40 µg/mL; ProSci [Cat. 5807]) were also included to detect epitope-tagged or native IFITM3 variants [7]. β-tubulin recognized by 1 µg/ml murine monoclonal anti-β-tubulin antibody (Sigma) was used as loading controls.

### Confocal microscopy

Subcellular localization of FL IFITM3, Δ21 IFITM3, and Y20A IFITM3 was measured by confocal microscopy. IFITM3-expressing A549 cells were washed twice with phosphate buffered saline (PBS) and fixed in 4% formaldehyde at 25°C for 20 minutes. Fixed cells were then permealized with 1% Triton X-100 (Sigma) for 20 min, blocked in 1% bovine serum albumin (BSA; Sigma) at 25°C for 1 hour, and labeled with primary antibodies for 2 hours or overnight. Primary antibodies used in this study included a murine anti-lysosomal-associated membrane protein 2 (LAMP2; H4B4 conjugate with Alexa 488, 1∶100 dilution; Santa Cruz Biotech) and a goat anti-IFITM2/3 (5 µg/mL; R&D Systems) antibodies. After primary antibody staining, cells were labeled with an anti-goat Alexa 633-conjugated secondary antibodies (1∶2000 dilution; Invitrogen) for 1 hour, counterstained with 4′,6-diamidino-2-phenylindole (DAPI; Thermo Scientific), and then analyzed by confocal microscopy using the Leica TCS-SP5 laser confocal imaging system (objective 63X).

## Results

### Epitope-tagged Δ21 IFITM3 restricts entry of IAV

**Figure 1:**
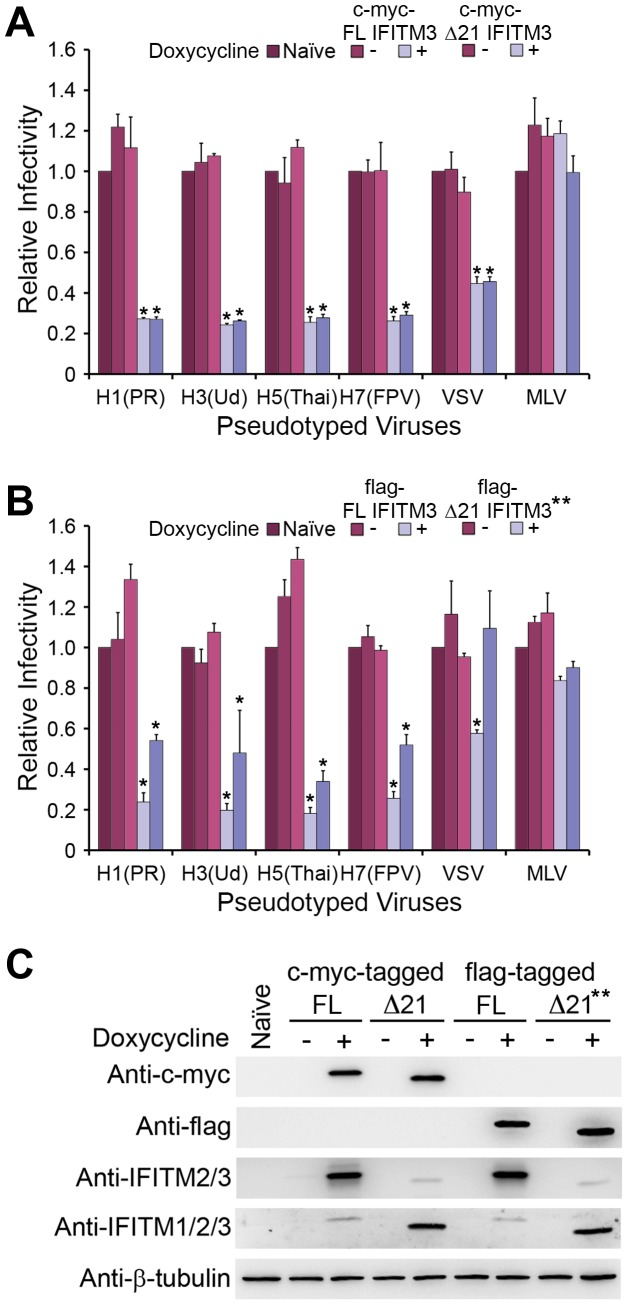
Epitope-tagged FL IFITM3 and Δ21 IFITM3 restrict IAVs. Naïve A549 cells and A549 cells transduced with tet-inducible c-myc-tagged (A), flag-tagged (B) FL IFITM3 or Δ21 IFITM3 were treated with or without 1000 ng/ml doxycycline (** 600 ng/ml doxycycline). Two days later, cells were infected with MLV-GFP pseudotyped with the indicated viral entry glycoproteins. Infected cells were harvested, fixed with formaldehyde, and analyzed by flow cytometry 48 hours after infection. The relative infectivity was determined as the percentage of GFP positive cells normalized to that of naïve A549 cells. (C) The same aliquots of cells used in (A) or (B) were analyzed by western blotting. The expression of IFITM3 isoforms was measured using the indicated antibodies. The expression of β–tubulin was used as our loading controls. The results are the averages of three independent infection replicates. The error bars indicate standard deviations. Each panel represents at least two sets of experiments with similar results. * indicates statistical significance (p<0.03 by Student's *t* test) as compared with naïve cells.

To characterize the effects of epitope-tagged FL IFITM3 and Δ21 IFITM3 on viral entry, A549 cells, a human lung adenocarcinoma epithelial cell line, were transduced with tet-inducible plasmids encoding FL IFITM3 or Δ21 IFITM3, respectively. Cells treated with or without doxycycline were incubated with MLV-GFP pseudotyped with IAV H1, H3, H5, and H7 proteins, with VSV G protein, or with MLV envelope (env) protein. As shown in Fig. 1A, N-terminal c-myc-tagged IFITM3 isoforms strongly restricted entry mediated by IAV H1, H3, H5, or H7 proteins and by VSV G proteins, whereas the inhibitory effects of flag-tagged Δ21 IFITM3 on IAV and VSV entry were attenuated (Fig. 1B). Entry of our control MLV pseudovirus was not affected by IFITM3 isoforms. To compare the inhibitory effects of both IFITM3 isoforms at similar protein expression levels, a lower concentration of doxycycline was used to induce the expression of flag-tagged Δ21 IFITM3. While we treated cells with the same concentration of doxycycline, we observed higher expression of flag-tagged Δ21 IFITM3 than that of FL IFITM3 (data not shown). The possible explanation is that more copies of the flag-tagged Δ21 IFITM3 gene might integrate into the chromosomes during initial transduction while we generated the stable cells.

The expression of epitope-tagged FL IFITM3 and that of Δ21 IFITM3 recognized by an anti-c-myc or an anti-flag antibody were comparable (Fig. 1C). The weaker signal for Δ21 IFITM3 detected by the anti-IFITM2/3 antibody, which interacts with the N-terminal 3-57 amino acids on IFITM3, might be due to its lower affinity for Δ21 IFITM3. By contrast, an anti-IFITM1/2/3 antibody, which recognizes the center region of IFITM proteins, and was used in previous research [7], interacted well with Δ21 IFITM3 but poorly with FL IFITM3 (Fig. 1C and Fig. S1A). Thus, the anti-IFITM1/2/3 antibody was no longer used in our subsequent experiments. (It has been shown that tyrosine 20 of IFITM3 regulates ubiquitination of FL IFITM3 [13]. Tyrosine 20-mediated posttranslational modifications might contribute to the weaker signal of FL IFITM3 detected by the anti-IFITM1/2/3 antibody.) Based on our studies, we observed that the restriction effects of Δ21 IFITM3 on IAV and VSV entry varied when different epitopes were used to tag IFITM3 isoforms. However, they provided us with useful information for comparing the expression levels of IFITM3 isoforms.

### Native Δ21 IFITM3 restricts IAV entry and replication

**Figure 2:**
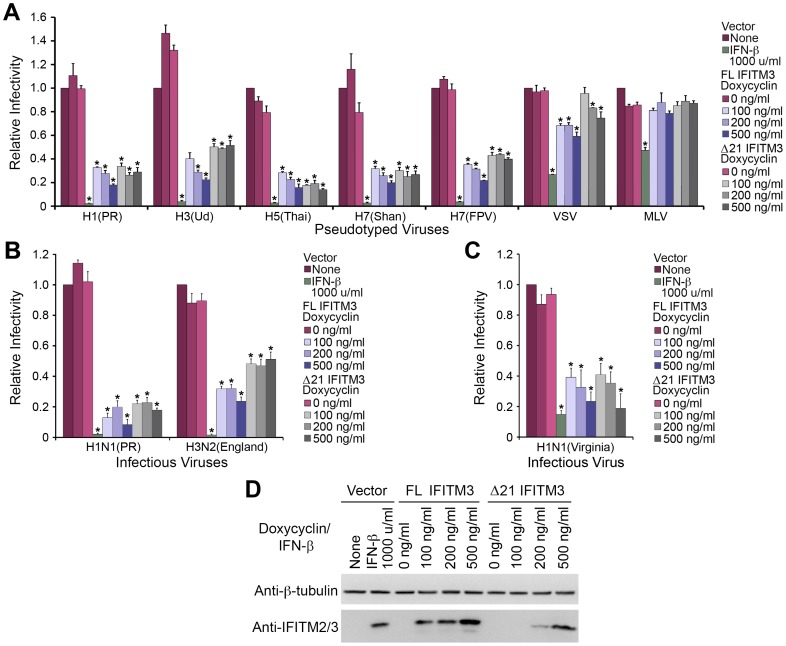
Native FL IFITM3 and Δ21 IFITM3 restrict IAVs. (A) A549 cells transduced with the vector alone or with tet-inducible native FL IFITM3 or Δ21 IFITM3 were treated with the indicated concentrations of doxycycline or interferon-β. Two days later, cells were infected by MLV-GFP pseudotyped with the indicated viral entry glycoproteins. Infected cells were harvested, fixed with formaldehyde, and analyzed by flow cytometry 48 hours after infection. The relative infectivity was determined as the percentage of GFP positive cells normalized to that of vector-transduced A549 cells. (B) The same aliquots of cells used in (A) were incubated with infectious influenza A/PR/8/34 (H1N1) virus at an M.O.I. of 1 or with influenza A/England/42/72 (H3N2) virus at an M.O.I of 0.5. Infected cells were labeled with anti-H1 or anti-H3 antibodies and with an PE-conjugated secondary antibody. Cells were analyzed by flow cytometry 16 hours after infection and the relative infectivity was determined as the percentage of PE positive cells normalized to that of vector-transduced A549 cells. (C) Experiments were similar to that in (B) except that cells were incubated with infectious influenza A/Virginia/ATCC3/2009 (H1N1) virus at an M.O.I. of 1. (D) The same aliquots of cells used in (A) or (B) were analyzed by western blotting. The expression of IFITM3 isoforms was measured using the indicated antibodies. The results are the averages of three independent infection replicates. The error bars indicate standard deviations. Each panel represents at least two sets of experiments with similar results. * indicates statistical significance (p<0.03 by Student's *t* test) as compared with vector-transduced cells.

Because of the inconsistent inhibitory effects of epitope-tagged IFITM3 isoforms on IAV and VSV entry, we sought to examine the function of native (non-tagged) FL IFITM3 and Δ21 IFITM3 in viral restriction. In our studies, we used A549 cells transduced with tet-inducible plasmids encoding native FL IFITM3 or Δ21 IFITM3, or with vector alone. MLV-GFP pseudotyped with H7 protein from influenza A/Shanghai/02/2013 (H7N9) virus, which caused an outbreak in China in 2013 [19], [20], was also included. Because the anti-IFITM2/3 antibody used in our studies had different affinities against FL IFITM3 and Δ21 IFITM3, we titrated their expression using different concentrations of doxycycline to examine their effects on IAV entry at comparable expression levels. As shown in Fig. 2A and Figs. S1B and C, in the presence of a low concentration of doxycycline, both native FL IFITM3 and Δ21 IFITM3 strongly restricted IAV H1-, H5-, and H7(Shan)-mediated entry. The inhibitory effect of Δ21 IFITM3 on entry of H3 and H7(FPV) pseudoviruses was weaker but still significant. In contrast to IAV, entry mediated by VSV G protein was moderately suppressed by FL IFITM3 and marginally affected by Δ21 IFITM3; these results are in agreement with previous observations [11]. Similar phenomena could also be observed in our viral replication assays. Replication of influenza A/PR/8/34 (H1N1) and A/Virginia/ATCC3/2009 (H1N1) viruses was suppressed efficiently by both IFITM3 isoforms, whereas influenza A/England/42/72 (H3N2) virus replicated more efficiently in Δ21 IFITM3-expressing than in FL IFITM3-expressing cells (Figs. 2B and C).

In our research, IFN-treated cells were included as our positive controls. As predicted, with comparable expression levels between IFN-induced and tet-induced IFITM3 (Fig. 2D), stronger suppression of viral entry and replication was observed in cells treated with IFN. This enhanced restriction effect could be attributed to expression of additional IFN-inducible effectors. Owing to the lack of an epitope tag and commercially available antibodies equally recognizing IFITM3 isoforms, expression of Δ21 IFITM3 was assayed using anti-IFITM2/3 antibody as described previously. Although weak signals for Δ21 IFITM3 could be detected, its expression should have been substantial and comparable to that of FL IFITM3. In addition, the expression levels of both IFITM3 isoforms were similar to that of IFITM3 induced by IFN. Previous studies have revealed the inability of Δ21 IFITM3 to restrict H1N1 IAV infections and the functional importance of the N-terminal 21 amino acids in IFITM-mediated restriction [7], [11], [12]. By contrast, our data demonstrated that both IFITM3 isoforms were functionally competent to restrict entry mediated by IAV H1, H5, and H7(Shan) proteins and to suppress replication of H1N1 IAV. The inhibitory effect of Δ21 IFITM3 on H3 and H7(FPV) infection was weaker than that of FL IFITM3 but still very prominent.

### Tyrosine 20 of IFITM3 is not a major determinant for IFITM-mediated restriction

**Figure 3:**
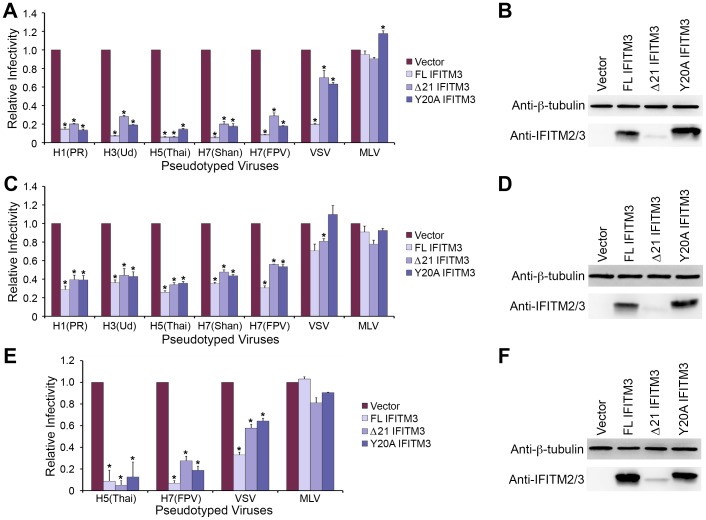
Y20A IFITM3 restricts IAV entry. A549 (A), 293T (C), or Vero E6 (E) cells transduced with vector alone or to express FL IFITM3, Δ21 IFITM3, or Y20A IFITM3 were incubated with MLV-GFP pseudotyped with the indicated viral entry glycoproteins. Infected cells were harvested, fixed with formaldehyde, and analyzed by flow cytometry 48 hours after infection. The relative infectivity was determined as the percentage of GFP positive cells normalized to that of vector-transduced A549 cells. Expression of the indicated IFITM3 variants in A549 (B), 293T (D), or Vero E6 (F) cells were detected by western blotting using the indicated antibodies. The results are the averages of three independent infection replicates. The error bars indicate standard deviations. Each panel represents at least two sets of experiments with similar results. * indicates statistical significance (p<0.03 by Student's *t* test) as compared with vector-transduced cells.

Tyrosine 20 of IFITM3 has been shown to be critical for IFITM3 endocytic trafficking and, therefore, plays an essential role in viral entry restriction [12]–[14]. According to our studies, Δ21 IFITM3 suppressed IAV entry and replication, indicating that tyrosine 20 may not be a major determinant for IFITM-mediated restriction. To examine this possibility, we generated an IFITM3 variant by replacing tyrosine with alanine (Y20A IFITM3). As shown in Figs. 3A and B, when we transiently hyperexpressed FL IFITM3, Δ21 IFITM3, and Y20A IFITM3 in A549 cells, entry of IAV was suppressed efficiently by each of the three IFITM3 variants. By contrast, Δ21 IFITM3 and Y20A IFITM3 slightly inhibited VSV infection, whereas MLV entry was not affected. In addition to A549 cells, entry restriction of IAV could similarly be observed in FL IFITM3-, Δ21 IFITM3-, or Y20A IFITM3-expressing 239T and Vero E6 cells (Figs. 3C to F). Because of their extremely low infectivity in Vero E6 cells, the effects of IFITM3 variants on IAV H1- and H3-mediated entry into Vero E6 cells were not evaluated. In summary, our data demonstrated that both Δ21 IFITM3 and Y20A IFITM3 restricted IAV entry, suggesting that tyrosine 20 of IFITM3 is functionally dispensable for IFITM3-mediated IAV restriction.

### Native IFITM3 and epitope- tagged IFITM3 variants have different subcellular distributions

**Figure 4:**
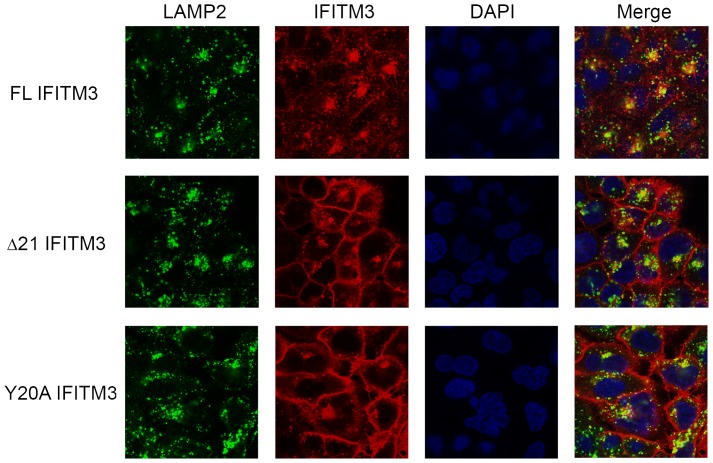
Δ21 IFITM3 and Y20A IFITM3 have boarder subcellular distributions. A549 cells expressing native FL IFITM3, Δ21 IFITM3, or Y20A IFITM3 were fixed, permeabilized, and labeled with anti-LAMP2 (green) and anti-IFITM2/3 (red) and DAPI (blue) antibodies. DAPI was used as nuclear counterstain. Cells were imaged by confocal microscopy. Each panel represents at least two sets of experiments with similar results.

**Figure 5:**
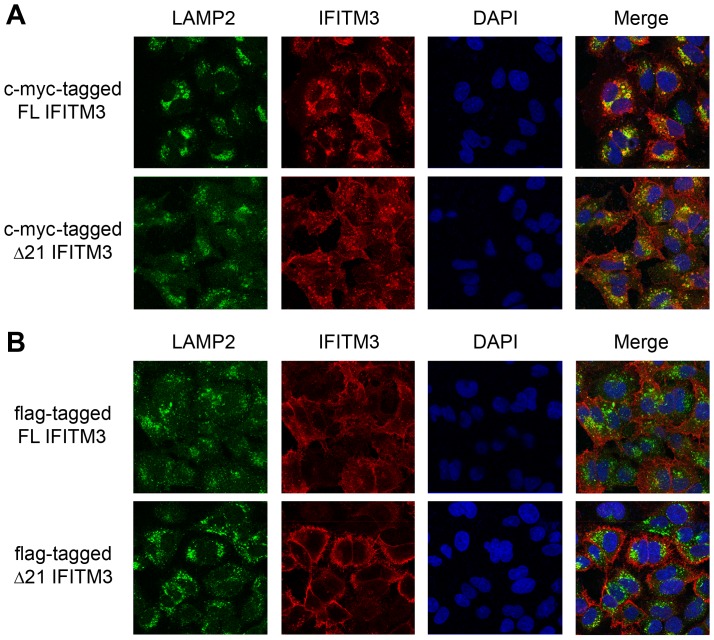
Epitope-tagged IFITM3 isoforms have altered subcellular distributions. A549 cells expressing the indicated c-myc- (A) or flag-tagged (B) IFITM3 isoforms were fixed, permeabilized, and labeled with anti-LAMP2 (green) and anti-IFITM2/3 (red) and DAPI (blue) antibodies. Cells were then imaged by confocal microscopy. Each panel represents at least two sets of experiments with similar results.

The subcellular localization of IFITM proteins was thought to be critical for their inhibitory effects on viral entry. Jia et al. showed that— compared with flag-tagged FL IFITM3, which distributes mainly in late endosomes/lysosomes—flag-tagged Δ21 IFITM3 localizes primarily to the periphery of the cell [11]. To examine whether native IFITM3 isoforms have similar subcellular distributions, confocal analysis was performed. We observed that native Δ21 IFITM3 and Y20A IFITM3 had broader subcellular distributions than FL IFITM3. Nevertheless, a large amount of Δ21 IFITM3 and Y20A IFITM3 still colocalized with LAMP2, a late-endosomal/lysosomal marker (Fig. 4). When epitope tags were added to the N-terminus of IFITM3 isoforms, their subcellular distribution was changed. Both c-myc-tagged IFITM3 isoforms concentrated in late-endosomal/lysosomal compartments (Fig. 5A), whereas a minimal amount of flag-tagged FL IFITM3 and Δ21 IFITM3 colocalized with LAMP2 (Fig. 5B). These diverse patterns of subcellular distributions were compatible with our viral entry data. Flag-tagged Δ21 IFITM3, which mainly distributed to the plasma membrane, had the weakest effect on viral entry. However, endosome/lysosome-associated native Δ21 IFITM3 and Y20A IFITM3 might contribute to efficient inhibition of IAV infection. The broader distributions of Δ21 IFITM3 and Y20A IFITM3 (compared with FL IFITM3) at both the plasma and endosomal/lysosomal membranes raises the possibility that the N-terminal 21 amino acids and tyrosine 20 play important roles but may not be the only determinants contributing to the subcellular localization of IFITM3. Because epitope tags altered the subcellular distribution and interfered with the function of IFITM3, native IFITM3 isoforms would be a better model for IFITM3-related studies.

## Discussion

In our research, we demonstrated that, like FL IFITM3, Δ21 IFITM3 and Y20A IFITM3 efficiently restrict infection of IAVs. Furthermore, we showed that native Δ21 IFITM3 and Y20A IFITM3 have broader subcellular distributions than FL IFITM3. However, a substantial amount of Δ21 IFITM3 and Y20A IFITM3 still associate with late endosomes/lysosomes. These results indicate that tyrosine 20 partially regulates IFITM3 trafficking, but neither N-terminal 21 amino acids nor tyrosine 20 of IFITM3 plays a critical role in IFITM-mediated restriction. Although the attenuated effect of Δ21 IFITM3 and Y20A IFITM3 on H3 and H7(FPV) infections could be observed, this inhibition was still significant and even more prominent than the effect of FL IFITM3 on VSV entry. Because several endocytic pathways contribute to IAV cellular entry [21]–[23], we could not exclude the possibility that various strains of IAVs may have minor differences in their endocytic trafficking. These differences may result in differential IAV restriction mediated by Δ21 IFITM3 and Y20A IFITM3.

Everitt et al., John et al., and Chesarino et al. have shown that Δ21 IFITM3 or Y20A IFITM3 loses its ability to restrict H1N1 IAV infection *in vitro* [7], [12], [13]. However, our data suggest that 21 amino acids at the N-terminus of IFITM3 are not essential for H1N1 IAV restriction. There are two possible reasons to explain these discrepancies. First, native or epitope-tagged IFITM3 isoforms were used in different studies [11]–[13]. As shown in our research, epitope tags interfered with the function and the subcellular distribution of IFITM3 isoforms, indicating that native IFITM3 may be a better system for IFITM3-realted studies. Second, expression of FL IFITM3 and Δ21 IFITM3 was not well controlled in previous research [7]. Everitt et al. used an anti-IFITM1/2/3 antibody, which interacts poorly with FL IFITM3 based on our observations, to recognize both IFITM3 isoforms [7]. The signals of FL IFITM3 and Δ21 IFITM3 detected by western blotting were comparable in this research suggesting that the actual expression level of FL IFITM3 should be multiple folds higher than that of Δ21 IFITM3. In our studies, we used various antibodies, native and epitope-tagged IFITM3 isoforms, as well as the tet-inducible system to evaluate the effects of FL IFITM3 and Δ21 IFITM3 on IAV entry at comparable protein expression levels. To assay the anti-viral activities of IFITM3 isoforms with appropriate expression, we further included IFN-treated cells as our controls. Metabolism of IFITM3 isoforms may be another variant contributing to different anti-viral activities of IFITM3 isoforms. However, we observed that FL IFITM3 and Δ21 IFITM3 have similar turnover rates (data not shown).

Tyrosine 20 of IFITM3 has been reported to be critical for regulating IFITM3 ubiqitination and its association with AP2 [13], [14]. Previous research has also suggested that AP2-mediated endocytic trafficking is essential for IFITM3 to restrict H1N1 IAV infection [14]. In contrast to previous observations, our data revealed that both Δ21 IFITM3 and Y20A were still competent to inhibit H1N1 IAV, indicating that tyrosine 20 is not a major determinant for IFITM3-mediated restriction. The maintenance of substantial levels of late endosome/lysosme-associated Δ21 IFITM3 and Y20A IFITM3 (in the absence of putative AP-2 interaction sites on IFITM3 variants) suggests that additional cellular factors, which are irrelevant to AP2-mediated endocytosis, may contribute to the trafficking of IFITM3 and efficient IAV restriction by both IFITM3 variants.

The association between *IFITM3* rs12252-C polymorphism and the severity of influenza is currently controversial. Everitt et al. and Zhang et al. observed increased morbidity and mortality in rs12252-C/C carriers during the H1N1 influenza pandemic, whereas this association could not be established by Mills et al. [7], [8], [24]. Owing to extremely small numbers of rs12252-C/C carriers recruited in all three studies, further analyses with large-scale cohorts are necessary to allow a conclusion to be drawn properly. However, according to our data, Δ21 IFITM3 restricted entry and replication of H1N1 IAV efficiently, thus suggesting that the poor clinical outcomes in rs12252-C carriers after H1N1 IAV infection may not be related to IFITM-mediated viral restriction. In our studies, we did observe that Δ21 IFITM3 had an attenuated but significant effect on restricting replication of H3N2 IAV. Whether *IFITM3* rs12252-C polymorphism is a prognostic factor for H3N2 influenza will require further clinical and epidemiological analysis.

Recent studies revealed that IFITM3 enhances survival of lung tissue-resident memory CD8^+^ T cells [25], indicating that IFITM proteins play some role in regulating adaptive immune responses. In addition, higher serum cytokine levels in rs12252-C carriers after avian influenza H7N9 infections could be detected [10], suggesting that IFITM3 may regulate cytokine/chemokine production. If future studies conclude that *IFITM3* rs12252-C polymorphism is associated with the severity of influenza, further investigations into immune reactions of subjects expressing different IFITM3 isoforms may provide greater insights into the underlying mechanisms.

## Supporting Information

Figure S1 (A) Naïve A549 cells or A549 cells transduced with tet-inducible native FL IFITM3 or Δ21 IFITM3 were treated with or without 1000 ng/ml doxycycline. Two days later, expression of IFITM3 isoforms was assayed by western blotting using the indicated antibodies. (B) A549 cells transduced with the vector alone or with tet-inducible native FL IFITM3 or Δ21 IFITM3 were treated with the indicated concentrations of doxycycline or interferon-β. Two days later, cells were infected by MLV-GFP pseudotyped with the indicated viral entry glycoproteins. Infected cells were harvested, fixed with formaldehyde, and analyzed by flow cytometry 48 hours after infection. The relative infectivity was determined as the percentage of GFP positive cells normalized to that of vector-transduced A549 cells. (C) The same aliquots of cells used in (B) were analyzed by western blotting. The expression of IFITM3 isoforms was measured using the indicated antibodies. The results are the averages of three independent infection replicates. The error bars indicate standard deviations. Each panel represents at least two sets of experiments with similar results. * indicates statistical significance (p<0.03 by Student's *t* test) as compared with vector-transduced cells.
(TIF)
