# Supplementary figures and images for: IFITM3 Polymorphism rs12252-C Restricts Influenza A Viruses
Source: PLoS One. 2014 Oct 14;9(10):e110096. doi: 10.1371/journal.pone.0110096 (PMC4196997; doi:10.1371/journal.pone.0110096)

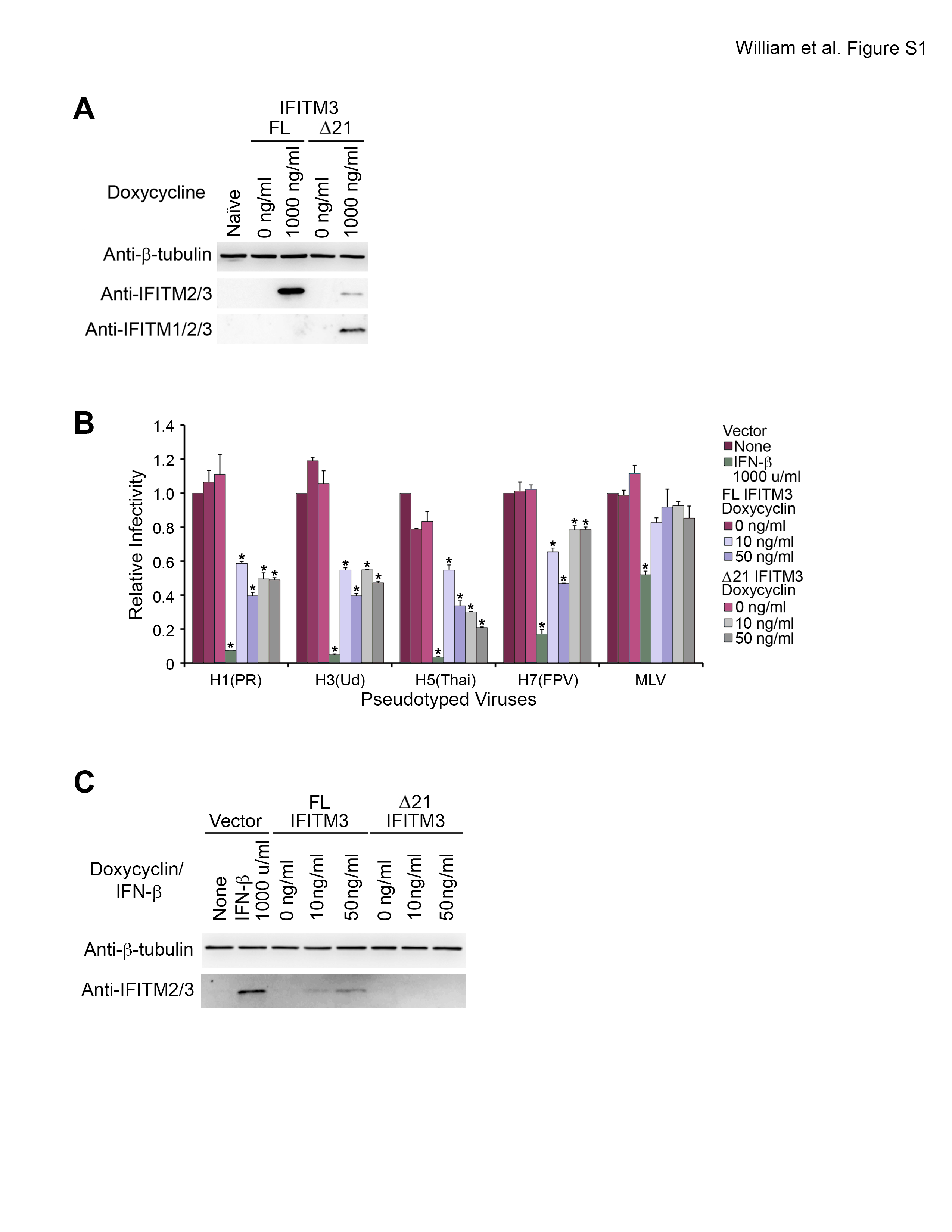

Supplement: Figure S1 — (A) Naïve A549 cells or A549 cells transduced with tet-inducible native FL IFITM3 or Δ21 IFITM3 were treated with or without 1000 ng/ml doxycycline. Two days later, expression of IFITM3 isoforms was assayed by western blotting using the indicated antibodies. (B) A549 cells transduced with the vector alone or with tet-inducible native FL IFITM3 or Δ21 IFITM3 were treated with the indicated concentrations of doxycycline or interferon-β. Two days later, cells were infected by MLV-GFP pseudotyped with the indicated viral entry glycoproteins. Infected cells were harvested, fixed with formaldehyde, and analyzed by flow cytometry 48 hours after infection. The relative infectivity was determined as the percentage of GFP positive cells normalized to that of vector-transduced A549 cells. (C) The same aliquots of cells used in (B) were analyzed by western blotting. The expression of IFITM3 isoforms was measured using the indicated antibodies. The results are the averages of three independent infection replicates. The error bars indicate standard deviations. Each panel represents at least two sets of experiments with similar results. * indicates statistical significance (p<0.03 by Student's t test) as compared with vector-transduced cells. (TIF) [file pone.0110096.s001.tif]
